# Challenges in achieving the UNAIDS 95-95-95 targets: evaluation of the antiretroviral therapy program among adult HIV/AIDS patients at Woldia General Hospital, Northeast Ethiopia

**DOI:** 10.3389/fpubh.2025.1524936

**Published:** 2025-06-16

**Authors:** Seada Seid, Mezgebu Yitayal, Lake Yazachew, Abdurahman Arega, Tilahun Dessie, Hassen Ahmed Yesuf, Jemila Seid Hassen, Abdulaziz Kebede Kassaw, Ali Yimer

**Affiliations:** ^1^Department of Public Health, College of Health Sciences, Woldia University, Woldia, Ethiopia; ^2^Department of Health Systems and Policy, College of Medicine and Health Sciences, Institute of Public Health, University of Gondar, Gondar, Ethiopia; ^3^Department of Management, College of Business and Economics, Woldia University, Woldia, Ethiopia; ^4^Department of Paediatrics and Child Health, College of Medicine and Health Sciences, Wollo University, Dessie, Ethiopia; ^5^Department of Biomedical Science, School of Medicine, College of Health Sciences, Woldia University, Woldia, Ethiopia; ^6^Department of Nursing, School of Nursing and Midwifery, College of Health Sciences, Addis Ababa University, Addis Ababa, Ethiopia; ^7^Department of Health Informatics, School of Public Health, College of Medicine and Health Sciences, Wollo University, Dessie, Ethiopia

**Keywords:** evaluation, process, antiretroviral treatment program, Ethiopia, HIV/AIDS

## Abstract

**Background:**

In Ethiopia and other resource-limited settings, antiretroviral therapy (ART) has been instrumental in reducing the harmful impact of the HIV pandemic. ART program aims to decrease morbidity and mortality, minimize healthcare costs, and enhance the quality of life for individuals living with HIV. However, the program faces several challenges, including poor medication adherence, a higher incidence of adverse effects, drug resistance, and persistent issues of discrimination and stigma. This study aimed to evaluate the implementation process of the antiretroviral therapy (ART) program among adult HIV/AIDS patients at Woldia General Hospital in Northeast Ethiopia.

**Methods:**

Between June 1 and June 30, 2020, a mixed-method case study was conducted. A total of 384 patient charts were reviewed, and 402 individuals living with HIV/AIDS were interviewed using systematic random sampling technique and interviewer administered questionnaire. Additionally, six key informant interviews were conducted, and 30 observations of healthcare professionals were recorded. The quantitative data were entered into Epidata version 4.6 and subsequently transferred to STATA version 14 for analysis. Bivariable analysis was performed to identify variables with *p*-values less than 0.2 as potential candidates for multivariable logistic regression, with statistical significance set at *p* < 0.05. The qualitative data underwent transcription, translation into English, coding, thematic analysis, and manual interpretation. The overall implementation status of the program was assessed based on predefined evaluation criteria.

**Results:**

According to national guidelines, 74.8% of the program was implemented consistently. Patient satisfaction was reported at 77%, healthcare personnel adherence to guidelines at 66%, and resource availability at 88.4%. Key challenges included shortages of test kits, Plumpy’Nut, and medications for opportunistic infections, as well as inadequate data management and issues with patient file handling. Most laboratory tests were not done according to the national guidelines. Additionally, refreshment training for health care providers was not given. Factors associated with patient satisfaction included the ability to disclose their condition (AOR = 3.25; 95% CI: 1.46, 7.26) and a waiting time of less than 30 min (AOR = 3.88; 95% CI: 1.26, 11.97).

**Conclusion:**

The overall level of program implementation was very good. However, patient satisfaction did not meet the national target of 85%, and adherence to national guidelines was minimal. Essential supplies such as HIV test kits, Plumpy’Nut, viral load tests, and medications for opportunistic infections were not consistently available. Additionally, most laboratory tests were not conducted in accordance with the guidelines. Factors associated with patient satisfaction included the ability to disclose HIV status and waiting times of under 30 min.

## Introduction

HIV has claimed approximately 42.3 million lives globally, making it a significant public health issue (1). Since the onset of the outbreak, around 75.7 million people have been infected with HIV. By 2019, approximately 25.4 million individuals were receiving antiretroviral treatment, and an estimated 38 million people were living with HIV globally. Additionally, there were about 1.7 million new HIV infections and 690,000 deaths attributed to AIDS-related causes (2). The first two cases of AIDS were documented in 1986, with HIV infection initially identified in Ethiopia in the early 1980s. Since then, the epidemic has rapidly spread across the country (3). Currently, the estimated national HIV prevalence rate in Ethiopia stands at 1.16% (4).

Following the introduction of the national antiretroviral (ARV) supply and use policy in 2002, Ethiopia became one of the first African countries to implement antiretroviral therapy (ART) in 2003, initially in a limited number of healthcare facilities (1). Providing high-quality healthcare is essential for preventing diseases, maintaining good health, and reducing illness and mortality to ensure national development (2). According to a 2018 UNAIDS report, Ethiopia had an adult ART coverage rate of 66% (3).

As a comprehensive program, ART aims to reduce healthcare costs for HIV-positive individuals while improving their Program at Woldia Hospitalg morbidity and mortality (5). It has been effective in achieving long-term suppression of HIV, reducing hospitalizations, mortality rates, AIDS-related complications, and opportunistic infections (OIs) (6).

Despite significant progress in combating AIDS as a public health threat, major gaps remain in service quality and accessibility (7). HIV/AIDS primarily affects the working population, impacting productivity in both agricultural and non-agricultural sectors, making it a significant barrier to Ethiopia’s overall development (8).

Ethiopia’s Federal Ministry of Health (FMOH) has acknowledged that disparities in health outcomes persist despite improvements in healthcare infrastructure, an increasing number of skilled healthcare professionals, and expanded access to medical services (9). A systematic review conducted in Ethiopia found that 15.3% of ART users experienced first-line treatment failure (10). Additionally, a meta-analysis indicated that the pooled incidence of second-line antiretroviral treatment failure was 5.98 per 100 person-years (95% CI: 4.32, 7.63) (11).

A study conducted in the north-eastern Ethiopian town of Hara found that only 71.8% of patients adhered to their ART regimen (12). Another study at Gondar University Hospital reported that for every 100 person-years, 12.26 HIV/AIDS patients were lost to follow-up (13).

The Antiretroviral Therapy (ART) Program at Woldia Hospital, Ethiopia, plays a crucial role in achieving several United Nations Sustainable Development Goals (SDGs) by improving health outcomes, reducing inequalities, and fostering socio-economic development. The evaluation of the ART program at Woldia Hospital provides insights into its effectiveness in reducing HIV/AIDS prevalence, improving treatment accessibility, and supporting socio-economic sustainability. By addressing these key issues, the program directly contributes to the achievement of multiple SDGs, particularly SDG 3 (Health), SDG 1 (Poverty), SDG 4 (Education), SDG 5 (Gender Equality), SDG 10 (Inequality Reduction), and SDG 17 (Partnerships) (14–16).

The 95-95-95 targets aim to ensure that:95% of people living with HIV know their HIV status,95% of people diagnosed with HIV are on ART and w95% of those on ART achieve viral suppression.

An evaluation of the ART program at Woldia Hospital would help measure progress toward these targets by:

Assessment of HIV testing and diagnosis: Evaluating how well the hospital identifies individuals with HIV through testing, aiming to ensure that 95% sub-dimensions of access to health care that defines the status (17). This involves analyzing the availability of HIV testing services and the number of people diagnosed.

Assessment of ART enrolment and adherence: Evaluating whether 95% of diagnosed individuals are being provided with ART. This includes monitoring ART initiation rates, retention in care, and adherence to treatment regimens (18).

Assessment of viral suppression: Evaluating the effectiveness of ART in achieving viral suppression, which is defined as a consistently low viral load (<200 copies/mL) among 95% of those on treatment. This is critical for preventing transmission and improving health outcome (19).

Through regular evaluations, Woldia Hospital can identify areas of improvement in diagnosis, treatment access, and retention. This supports the broader Ethiopian and global effort to meet the 95-95-95 targets. It also enables healthcare professionals to develop targeted interventions to increase the proportion of individuals who know their HIV status, are on ART, and achieve viral suppression, thus contributing to the broader goal of ending the HIV/AIDS epidemic.

To my knowledge, there have been few assessments of Ethiopia’s adult ART program implementation, despite the challenges it faces. Therefore, the findings of this evaluation could help the federal Ministry of Health and other governmental and non-governmental organizations prioritize the ART program. This research has contributed to understanding the implementation status of the ART program at Woldia General Hospital.

The effectiveness of treatment depends on the proper administration of clinical care components as outlined in the guidelines, making it essential. Therefore, regardless of whether the ART service is assessed based on these guidelines, the insights gained will be highly valuable for its provision and improvement. Moreover, process evaluation helps in understanding the key factors influencing outcomes. This study focused on evaluating the implementation process of the antiretroviral therapy (ART) program for adult HIV/AIDS patients at Woldia General Hospital in Northeast Ethiopia.

### Evaluation questions

Does Woldia Hospital have all necessary resources to provide HIV/AIDS care services? If yes, How and if no, why?

Do health care workers perform according to the national guideline in giving ART services? If yes, how and If no, Why?

Are people living with HIV/AIDS satisfied with the ART service?

### Evaluation objectives

#### General objective

To evaluate ART services implementation provided by health care workers in Woldia General Hospital, North East, Ethiopia, 2020

#### Specific objectives

To assess the availability of resources required to provide ART care services in Woldia Hospital, North East, Ethiopia, 2020

To assess adherence/compliance of health care workers with the national ART guide line in delivering ART care services at Woldia Hospital, North East, Ethiopia, 2020

To determine the level of satisfaction of HIV/AIDS patients toward ART care services provided by Health care workers, North East, Ethiopia, 2020

To identify barriers to ART services implementation at Woldia Hospital, North East, Ethiopia, 2020

To identify factors that determine satisfaction of HIV/AIDS patients toward ART care service, North East, Ethiopia, 2020

### Evaluation methods

#### Evaluation design and setting

A case study design with a mixed-methods approach was employed to evaluate the process of ART program’s implementation among adult HIV/AIDS patients at Woldia General Hospital, Ethiopia, from June 1 to 30, 2020. Both qualitative and quantitative data were collected simultaneously. The hospital serves over 2 million residents from the town and surrounding woredas and has been offering ART services since 2005. Currently, 4,215 HIV/AIDS patients, including 4,021 adults and 194 children, are registered at the ART clinic for treatment.

#### Evaluation approach

Formative evaluation was done to assess implementation of ART program in Woldia Hospital. Formative evaluation was conducted for the purpose of improving the program by identifying the gaps.

#### Focus of evaluation

This evaluation was focused on implementation status of ART program; that was how ART was implemented and why if it was not implemented, what works? To improve the program what was done? For understanding implementation of ART program delivery in line with the national ART guideline. So, the evaluation understanding, describing, testing, and improving components of a programs process theory; that includes program organizational plan (resources to be used and activities to be performed) and services utilization plan (assumptions taken by the program about uptake of services produced, value given by target population) of the program logic model.

#### Dimension of evaluation

*Availability* is one of the five sub-dimensions of access to health care that defines the relationship between the volume and types of services and resources to the client’s volume. Availability measures the structural elements like infrastructure, human resources, guidelines, supplies and drugs. Structure viewed as the capacity to provide high quality care. It refers to the characteristics of the setting in which care takes place including program inputs or efforts that enhance the health facility readiness to provide the intended services when clients came for the required services. These factors control how providers and patients in a healthcare system act and are measures of the average quality of care within a facility or system.

*Satisfaction:* used to measure immediate outcome of the service and it deals with the fitness between services and clients need.

*Compliance:* is one of the criteria to measure implementation and it is the degree to which ART care services being implemented in Woldia Hospital according to the national guidelines and clinical parameters and protocols.

#### Study populations

The study population included HIV/AIDS patients receiving care and making multiple hospital visits, ART patient records with at least 6 months of follow-up, healthcare providers available during data collection, and administrative staff and team leaders who had worked at the hospital for more than 6 months.

#### Sample size and sampling procedures

To evaluate patient satisfaction, the required sample size was determined using a proportion of 57.6% (*p* = 0.57), based on a study conducted in Wollega, which reported that 57.6% of patients were satisfied with ART care services (20).

The margin of error (d) was set at 5% (0.05) with a 95% confidence level. Using the single population proportion formula:

$$n=(Z\;\alpha /2)2^{*}\mathrm{pq}/\mathrm{d2}=(1.96)2(0.57^{*}(1-0.57))/(0.05)2$$

$$=3.84^{*}0.24/0.0025$$

=377

To account for a 10% non-response rate (38 participants), the final sample size was adjusted to 415.

Systematic random sampling technique was employed to select study participants to measure satisfaction. Total adult HIV/AIDS patients receiving care currently at Woldia Hospital were around 4,021 and around 60 HIV/AIDS patients receives care per day. From those K was determined by (Sample size needed 415, K=N/n = 4021/415 = 9) and by taking lottery method from (1-9) the starting unit was determined and using systematic random sampling technique study participants were selected every nine interval based on the ordering of coming until the sample size was 415.

#### Document review

The document review included an assessment of ART registration books, patient charts, and administrative records/reports. A patient chart review was conducted to evaluate healthcare workers’ compliance with ART program implementation. Based on the available information, no prior evaluation had been conducted on healthcare workers’ adherence to adult ART service guidelines.

Assuming that 50% of clients received services in accordance with the national guidelines (*p* = 0.5), with a 5% margin of error and a 95% confidence interval, the single population proportion formula was applied:

$$n=(\mathrm{Z\alpha}/2)2^{*}\mathrm{pq}/d2$$

$$=(1.96)2^{*}0.5^{*}0.5/(0.05)2$$

=384

Total adult patients following ART at Woldia Hospital were around 4,021. Using simple random sampling technique by using computer generated lottery method 384 charts were selected and reviewed.

#### Key informant interviews

A total of six key informant interviews were conducted, involving team leaders and administrative staff.

The purpose of conducting key-informant interview with administrative staff and team leaders is to get detail information regarding program management and barriers to services implementation and solutions. Major issues to be addressed includes resource allocation and management, support system, barriers to service implementation from team leaders and administrative staff perspective.

#### Observations

A total of 30 observations were conducted to assess provider-client interactions and adherence preparation. These included physician-client interactions, nurse-client interactions, and adherence unit preparations. In each category, 10 observations were conducted, with the first and last three being excluded. In total, 30 observations were analyzed to assess healthcare workers’ compliance.

#### Variables and measurements

Indicators and measurements were developed based on the Ethiopian ART guidelines and relevant literature, ensuring the evaluation’s relevance and usefulness in assessing service status and identifying areas for improvement (3, 21–25). A formative evaluation approach was used to examine the implementation of the adult ART program. To assess the availability of essential drugs and medical equipment required for the program, eight indicators were utilized. Additionally, healthcare providers’ adherence to guidelines was evaluated using 16 indicators. Patient satisfaction with the service was measured through 15 indicators, each rated on a five-point Likert scale (1 = very dissatisfied, 2 = dissatisfied, 3 = neutral, 4 = satisfied, 5 = very satisfied). Patients who scored above 52% were classified as satisfied, based on the demarcation formula (26).

The process consists of a series of interconnected activities that contribute to attaining the programs objectives. Dimensions represent crucial aspects of the program that the investigator aims to assess. The weight of indicators refers to the importance assigned by stakeholders to each selected indicator before the evaluation, and the scores for these indicators were computed using a specified formula. The indicator score was determined according to the provided formula.

$$\text{Indicator score}=\frac{\text{Observed number}*\text{Indicator weight}}{\text{Expected number}}$$

Indicator weight = is the weight given by Stakeholders before the evaluation was done. Achievement (A) = Indicator score/weight*100. The indicator scores were calculated based on preset judgment criteria >85% = excellent (successfully implemented), 70-85% = very good (needs improvement), 55-69% = good (needs urgent improvement), 40-54% = fair(needs urgent and major improvement), <40% = critical (not implemented) (needs urgent and major improvement and modification) (25).

*Satisfaction:* Patients care received from ART service delivery points and staff captured through the five point likert scale (very dissatisfied = 1, Dissatisfied = 3, Neutral = 3, Satisfied = 4, Very satisfied = 5). It was measured using 15 item indicators and categorized using demarcation formula ≤52 as dissatisfied and > 52 as satisfied.

Over all highest score - over all lowest score + total lowest score (26).

*Drug adherence;* also called medication adherence which means taking HIV medicines every day and exactly as prescribed.

*Good drug adherent:* if the average adherence level was ≥ 95% or ≤3 doses missed per month (4).

*Fair drug adherent*: 85–94% or 4–8 doses missed per month (4).

*Poor drug adherent*: less than 85% or ≥ 9 doses missed per month and try to assess by asking the missed doses with-in the past 3 days, within 1 week and within 1 month (4).

*Process:* it means activities that can be performed in ART clinic to treat HIV/AIDS patients.

*Logic model:* a visual and systematic way that presented the relations between ART Intervention and its effects. It includes resources needed to make program operational, planned activities, and result that the program intended to achieve.

*Stakeholders:* are individuals, groups or organizations having a significant interest in how well ART program functions for instance those with decision-making authority over the program, funders, sponsors, administrators, personnel and clients or intended beneficiaries.

*Compliance*: It is used to measure implementation and it is the degree to which services being implemented according to the national ART guidelines and clinical parameters and protocols.

*Waiting time*: the time between the clients’ arrival at the service delivery points and the time the client received the health service (27).

#### Data collection tools and procedures

Structured and semi-structured questionnaires were used for the survey and key informant interviews, respectively, adapted from various evaluations and studies (3, 21–25). The questionnaires were initially developed in English, then translated into Amharic, the local language, and back into English to ensure consistency. For data collection and supervision, two BSc nurses and one public health professional were recruited. Additionally, a two-day training session was conducted to familiarize them with essential data collection and supervision techniques.

A pre-test was conducted at Dessie Referral Hospital with 21 HIV/AIDS patients, and the final tools were revised based on the pre-test findings. Supervisors monitored the data collection process daily, ensuring accuracy, consistency, and completeness. To minimize the Hawthorne effect, the first and last three observations were excluded. The qualitative data were then analyzed based on participants’ responses.

#### Data management and analysis

For quantitative data, completeness and consistency checks were performed before being coded by the supervisor and principal investigator. Data entry was done using Epi Data version 4.6, with daily checks to identify and correct missing values and errors. Binary logistic regression analysis was conducted, and variables with a *p*-value of less than 0.2 in the bi variable logistic regression analysis were considered for multivariable logistic regression. In the multivariable analysis, factors associated with satisfaction were identified using a p-value of less than 0.05 and an adjusted odds ratio (AOR) with a 95% confidence interval (CI).

For qualitative data, field notes were taken and refined into fair notes each night by the principal evaluator. Key informant interviews were audio-recorded, transferred to a computer, transcribed in Amharic, and later translated into English. Additionally, non-participatory observations were conducted to assess interactions between healthcare workers and patients using a structured questionnaire. Thematic analysis was applied to qualitative data, evaluating three key dimensions: resource availability, healthcare worker compliance, and patient satisfaction. Finally, the implementation level of the ART program was assessed based on predetermined judgment criteria for each dimension.

## Results

### Availability of resources

The ART service was provided in a dedicated unit. The hospital had trained professionals for ART services, including mentorship, consisting of one internist, three general practitioners, one health officer, six nurses, five pharmacists, four laboratory technicians, four health informatics professionals, and eight case managers. Currently, the ART unit is staffed by one general practitioner, three clinical and two BSc nurses, two laboratory professionals, two pharmacy professionals, four health informatics professionals, and all eight case managers.

The hospital offered various laboratory tests, including AFB, Gene-Xpert, and culture for TB diagnosis, stool examination, HBV and HCV tests, and blood film analysis. Additionally, HIV/AIDS-related tests such as CD4 counts, liver function tests, renal function tests, hematologic tests, and cervical cancer screening were available. At present, most of the laboratory tests required by the national ART implementation guidelines are accessible, except for the HIV test, the cryptococcal antigen test, and the viral load test. The viral load test was conducted by sending blood samples to Dessie Referral Hospital.

There was a shortage of HIV test kits due to their national expiration, resulting in the unavailability of HIV testing at the time of data collection. Additionally, some laboratory tests, such as CD4 counts, HBV and HCV tests, and cervical cancer screenings, faced interruptions due to a shortage of reagents.

Four of the key informant said there was no HIV test kit now because it was nationally expired.

Regarding drug availability, all essential medications, including ART drug regimens, CPT, INH, and anti-TB drugs, had been consistently available over the past 3 months. The hospital receives these medications regularly every 3 months from PFSA. However, there was a supply disruption for other essential drugs used for opportunistic infection (OI) treatment, such as fluconazole, acyclovir, miconazole, and clotrimazole. Additionally, shortages were reported for other medications, including cloxacillin and ciprofloxacin.

Two of the key informants said there is a shortage of opportunistic disease treatment drugs like fluconazole, acyclovir, miconazole, and clotrimazole, and currently they are not available. Previously, these drugs were charge-free. But now a day, it is not free. Previously, there were NGOs that supported these drugs for free, but now these organizations have been phased out.

Regarding infrastructure, the ART clinic had an onsite pharmacy, an examination room, a waiting area, an inpatient room, and a private counseling room. However, there was no onsite laboratory.

In terms of the ART pharmacy, it had secure storage space, pharmacy report forms, prescription forms, models, a pharmacy registration book, and a stock card. However, a bin card was not available, and there was no private counseling room within the pharmacy. Additionally, the ART examination rooms were found to be insufficient in size.

Two of the key informants said the ART service room was too narrow for two health professionals to treat patients in one room.

Comprehensive HIV services were provided, including voluntary counseling and testing (VCT), prevention of mother-to-child transmission (PMTCT), provider-initiated testing and counseling (PITC), tuberculosis (TB) clinic services, sexually transmitted infection (STI) management, opportunistic infection (OI) treatment, nutritional support, psychosocial assistance, and palliative care. Essential resources, such as the 2017 national guideline, ART prescriptions, referral slips, feedback forms, and recording and reporting documents, were consistently available.

Regarding monitoring and feedback mechanisms, a multidisciplinary team (MDT) conducted monthly supportive supervision and performance assessments of the ART service. Additionally, the regional health bureau and the Centers for Disease Control and Prevention (CDC) carried out quarterly evaluations (Tables 1, 2).

**Table 1:** Availability of resources for process evaluation of ART program among adult HIV/AIDS patients at Woldia Hospital, 2020.

S.no	Item	Yes	No	Remark available in the last 3 months
		Number		
1	ART guide line	1		
2	Availability of five first line ART drugs(TDF + 3TC + EFV, TDF + 3TC + DTG, TDF + 3TC + NVP, AZT + 3TC + EFV, AZT + 3TC + NVP)	✓		✓
3	Availability of 5 s line ART drugs(TDF + 3TC + ATV/RTV, TDF + 3TC + LPV/RTV, ABC + 3TC + LPV/RTV, AZT + 3TC + LPV/RTV, AZT + 3TC + ATV/RTV)	✓		✓
	Availability of two third line ART drugs(AZT + 3TC + DTG + DRV/RTV, TDF + 3TC + DTG + DRV/RTV)	✓		✓
4	CPT	✓		✓
5	INH	✓		✓
6	Trained health professionals including mentorship			
	GP	3		
	Specialist	1		
	Health officer	1		
	Nurse	6		
	Pharmacy	5		
	Laboratory	4		
	Data clerk	4		
	Case managers	8		
7	CD4 count test (machine)	1		
8	Viral load test	X		
9	CBC test	✓		
10	Chemistry test	2(chemistry machine)		
11	OI drugs (Acyclovir, fluconazole, miconazole Ketoconazole)	X		
12	Plumpinut	X		
13	Cryptococal antigen test	X		
14	Gene expert	✓		
15	AFB	✓		
16	X-ray	✓		
17	Stool examination	✓		
18	Microscope	✓		
19	HIV test kit	X		
20	Blood film	✓		
21	HBV test	✓		
22	HCV test	✓		
23	Pregnancy test	✓		
24	Pap smear	✓		
		✓		

**Table 2:** Summary of ART resource availability indicators at Woldia General Hospital, Ethiopia, 2020.

Indicators		W	O	E	S	A
Availability of trained health care workers		3	14	14	3	100%
Availability of five first line drugs regimens		5	5	5	5	100%
Availability of 5 s line drug regimens		5	5	5	5	100%
Availability of two third line drug regimens		5	2	2	5	100%
Availability of 2017 National ART guide line at least one		1	1	1	1	100%
Number of essential Laboratory test present	Viral load machine(x)	5	4	6	3.3	67%
	CD4 machine (1)					
	CBC machine (2)					
	OFT(chemistry machine)2					
	X-ray machine (1)					
	HIV test kit(x)					
Availability of comprehensive HIV care service		2	9	9	2	100%
Availability of essential OI drugs		4	2	5	1.6	40%
	Anti TB drugs including INH prophylaxis(yes)					
	Cotrimoxazole(yes)					
	Fluconazole(X)					
	Ketoconazole(X)					
	Acyclovir (x)					
Over all availability of resources for the ART program		30			25.9	88.4%(excellent)

W, Indicator weight; S, Indicator score; O, Observed value; E, Expected value; A, Achievement.

### Compliance

#### Background and clinical characteristics of study subjects for chart review

The majority of the participants were female, accounting for 61.5% of the total. The average duration of treatment was approximately 8 ± 3.8 years. Most patients (312; 81.2%) were receiving first-line treatment, and about 332 (86.4%) initiated treatment immediately after being deemed eligible. Nearly 90% of patients demonstrated good adherence to their medication (Table 3).

**Table 3:** Socio-demographic and clinical characteristics of clients from chart review at Woldia General Hospital, Ethiopia, 2020.

Variable	Category	Number	Percent (%)
Sex	Male	148	38.5
	Female	236	61.5
	Total	384	100
Age	18–25	34	8.85
	26–35	162	42.2
	36–45	117	30.5
	46–70	71	18.5
	Total	384	100
Duration of treatment	0.5–5 years	115	29.95
	6–10 years	160	41.67
	>10 years	109	28.39
	Total	384	100
Drug regimen	First line	312	81.25
	Second line	66	17.19
	Third line	6	1.56
	Total	384	100
Most recent CD4 count	≤ 200	48	13.6
	>200	305	86.4
	Total	353	100
Adherence status	Good	345	89.84
	Fair	23	5.99
	Poor	16	4.17
	Total	384	100

#### Compliance of HIV clinical care with national guideline

Among the 384 patient charts reviewed in this study, 301 patients met the eligibility criteria for cotrimoxazole preventive therapy (CPT) based on national guidelines. However, only 248 (82.4%) were receiving CPT, and among those on CPT, adherence was assessed in 88.6% of cases. Discontinuation of CPT was appropriately done in 99.6% of cases when patients no longer met the eligibility criteria.

Of the 306 patients whose ART regimen was changed, documentation of the reason for the change was available for 234 (76.4%). Among the 70 patients on second-line treatment, 65 (93%) had their regimen changed at the appropriate time. However, among the six patients receiving third-line therapy, only two (22%) underwent timely regimen changes. Baseline CD4 count was determined for 90% of patients, and 237 (61.7%) had CD4 testing conducted every 6 months.

Regarding viral load monitoring, only 28 (20.6%) of the 136 patients diagnosed with HIV from 2016 onward had their initial viral load measured within 6 months. Among 362 patients eligible for annual viral load testing, only 153 (42.26%) underwent yearly testing. ART drug adherence was assessed at every visit for 99.5% of patients.

Baseline screening assessments revealed that 108 (28%) and 162 (42%) of patients were tested for diabetes mellitus (DM) and hypertension (HTN), respectively. Hematocrit (HCT) was performed in 371 (96.6%) patients, and urine analysis was done for 314 (82%). Additionally, baseline renal function tests (RFT) and liver function tests (LFT) were conducted in 362 (94%) cases. Hepatitis B virus (HBV) and hepatitis C virus (HCV) screenings were performed in 81 (21%) and 42 (11%) of patients, respectively.

All patients were assessed for drug side effects, and 366 (95%) were screened for malnutrition at every visit. Among 106 malnourished patients, only 60 (56.6%) received treatment. Nearly all patients were screened for opportunistic infections and categorized based on WHO clinical staging. Among 177 patients eligible for cervical cancer screening, only 59 (33.3%) underwent screening. All patients diagnosed with HBV were on a tenofovir disoproxil fumarate (TDF)-based regimen.

However, none of the patients with a CD4 count below 100 were screened for cryptococcal antigen. Among 339 patients who had ever been on a TDF regimen, only 142 (42%) had blood pressure (BP) or glomerular filtration rate (GFR) measurements. For those who had ever been on an AZT regimen, 99.5% were evaluated for anemia at baseline, but only 68 (34%) were assessed for anemia at the fourth week of AZT initiation.

Among 363 patients eligible for isoniazid preventive therapy (INH), only half were receiving it. Nearly all patients were screened for tuberculosis (TB) at every visit. Among those who had been treated for TB, approximately 98% were on an appropriate ART regimen.

Data completeness was observed in 74% of cases. Adherence to follow-up schedules per national guidelines (at week 0, week 1, week 2, week 4, week 8, week 12, week 16, and week 24 after ART initiation) was maintained in 81.5% of patients. Among 100 patients who had been lost to follow-up, 65 (65%) eventually returned to treatment (Table 4).

**Table 4:** Summary of compliance indicators to assess the implementation level of ART program among adult HIV/AIDS patients at Woldia General Hospital, Ethiopia, 2020.

Indicator	W	O	E	S	A (%)
Proportion of HIV/AIDS patients eligible for CPT and on CPT	5	248	301	4.1	82
Proportion of HIV/AIDS patients who are eligible and on INH	5	182	363	2.5	50
Proportion of HIV/AIDS patients CD4 count was done every 6 month	2	237	384	1.2	60
Proportion of HIV/AIDS patients initial or baseline viral load was done	2	28	136	0.4	20
Proportion of HIV/AIDS patients viral load was done Q year	3	153	362	1.3	43.34
Proportion of HIV/AIDS patients who had baseline U/A	2	314	384	1.6	80
Proportion of HIV/AIDS patients who had baseline CBC	2	371	384	1.9	95
Proportion of HIV/AIDS patients who had baseline OFT	2	362	384	1.9	95
Proportion of HIV/AIDS patients assessed for malnutrition	5	366	384	4.7	94
Proportion of malnourished HIV/AIDS on nutritional therapy	5	60	106	2.8	56
Proportion of female eligible HIV/AIDS patients who were screened for cervical cancer	2	59	177	0.6	30
Proportion of HIV/AIDS patients who were assessed for drug adherence every visit	3	382	384	2.9	96.67
Proportion of HIV/AIDS patients who were screened for TB every visit	2	383	384	1.9	95
Proportion of HIV/AIDS patients who had complete address documented	3	284	384	2.2	73.34
Proportion of HIV/AIDS patients who screened for HBV	2	81	384	0.4	20
Proportion of HIV/AIDS patients who were lost and returned to treatment	5	65	100	3.2	64
	50			33.6	66(good)

W, Indicator weight; O, Observed value; E, Expected value; S, Indicator score; A, Achievement.

### Level of satisfaction of HIV/AIDS patients toward ART service

#### Socio- economic and demographic characteristics of study participants

In this evaluation, a total of 402 HIV/AIDS patients participated, achieving a response rate of 97%. The median age of participants was 36 years (IQR: 12), with ages ranging from 18 to 70 years. A significant portion, 168 (41.8%), fell within the 26–35 age group, and 221 (55%) were female. The majority, 273 (68%), were married, while 173 (43.3%) had completed secondary education. Most participants, 322 (80.1%), resided in urban areas, and 266 (66.2%) were living with their partner (Table 5).

**Table 5:** Socio-demographic characteristics of HIV/AIDS patients at Woldia General Hospital, Ethiopia (*n* = 402).

Variables	Category	Frequency (%)
Age	18–25	32(0.08)
	26–35	168(41.8)
	36–45	134(33.34)
	≥ 46	68(16.9)
Sex	Male	181(45.02)
	female	221(54.98)
Residence	Urban	322(80)
	Rural	80(20)
Marital status	Married	281(69.9)
	Single	74(18.4)
	Divorced	19(4.7)
	Widowed	28(6.96)
Educational status	No formal education	85(21.14)
	Primary school	100(24.87)
	Secondary school	173(43.03)
	Diploma and above	44(10.94)
Occupational status	Unemployed	45(11.2)
	Government employed	48(11.94)
	Merchant	48 (11.94)
	Farmer	46(11.44)
	Daily labor	59(14.67)
	Driver	20(4.97)
	House wife	117(29.1)
	Student	19(4.72)
Level of income in ETB	500–1200	123(30.6)
	1,201–2400	135(33.6)
	2,401–8000	144(35.8)
Living with	Partner	266(66.16)
	Family	80(19.9)
	Alone	48(11.94)
	Friend	8(1.99)

#### Clinical characteristics of the study participants for satisfaction from chart review

The evaluation showed that 332 (82.6%) of the respondents were on a first-line regimen, and 331 (82.34%) were at recent WHO clinical stage I. 353 (87.81%) had a CD4 count greater than 200. The study also showed that 154 (38.0%) had been on ART from 6 to 10 years, and 368 (91.5%) disclosed their HIV status (Table 6).

**Table 6:** Clinical characteristics of the study participants at Woldia General Hospital to asses’ satisfaction of HIV/AIDs patients toward the ART service, Ethiopia, 2020.

Variables	Category	Frequency (%)
ART regimen	First line	332 (82.58)
	Second line	67 (16.67)
	Third line	3 (0.75)
WHO stage	Stage I	331(82.34)
	Stage II	43(10.69)
	Stage III	16(3.98)
	Stage IV	12(2.98)
Recent CD4 count	≤200	49(12.19)
	>200	353(87.81)
Duration on ART	≤ 5Yr	144(35.82)
	6–10Yr	154(37.81)
	11–16Yr	104(25.87)
Disclosure status	Yes	368(91.54)
	No	34(8.5)
CD4 done every 6 month	Yes	298(74.12)
	No	104(25.87)
Waiting time in minute	10–29	146(36.31)
	30–60	235(58.45)
	61–120	21(5.22)

### Level of satisfaction

The overall satisfaction rate with antiretroviral services was 77%, with a 95% confidence interval (CI) of 73.5–81.7%. Satisfaction levels for specific indicators varied between 62.2 and 88.5%.

In general, the highest satisfaction was reported for healthcare workers’ competency (88.5%) and confidentiality and privacy during examinations (85%). However, lower satisfaction levels were observed regarding chart handling (62.5%) and the availability of opportunistic infection (OI) drugs (Table 7).

**Table 7:** Satisfaction indicators for process evaluation of ART program among adult HIV/AIDS patients at Woldia General Hospital, Ethiopia, 2020.

Indicator	W	O	E	S	A
Proportion of ART clients who satisfied with the adherence counseling service	2	345	402	1.7	85%
Proportion of ART clients who satisfied with the hospital cleanness	1	333	402	0.83	83%
Proportion of ART clients who satisfied with the appointment date/time	1	330	402	0.82	82%
Proportion of ART clients who satisfied with the competency of health care workers	2	356	402	1.77	88.5%
Proportion of ART clients who satisfied with the courtesy of health care workers	1	334	402	0.83	83%
Proportion of ART clients who satisfied with the pharmacy service	2	314	402	1.56	78%
Proportion of ART clients who satisfied with the laboratory service	2	314	402	1.56	78%
Proportion of ART clients who satisfied with the examination classes	1	280	402	0.69	69%
Proportion of ART clients who satisfied with the place where ART service unit found	1	327	402	0.81	81%
Proportion of ART clients who satisfied with service hour of the hospital	1	283	402	0.7	70%
Proportion of ART clients who satisfied with the availability of OI drugs	1	279	402	0.69	69%
Proportion of ART clients who satisfied with the confidentiality and privacy during examination	1	345	402	0.85	85%
Proportion of ART clients who satisfied with the waiting time	1	286	402	0.71	71%
Proportion of ART clients who satisfied with the waiting area	1	285	402	0.7	70%
Proportion of ART clients who satisfied with chart handling	1	250	402	0.62	62%
O	20			14.84	77%(very good)

E, Expected; O, Observed; W, Weight; A, Achievement; S, Indicator score.

### Factors associated with satisfaction

In this evaluation, two factors were found to be associated with patient satisfaction: the ability to disclose sero-status and a waiting time of less than 30 min. Patients who disclosed their sero-status had 3.25 times higher odds of being satisfied (AOR = 3.25; 95% CI: 1.46–7.26) compared to those who did not. Similarly, those who waited less than 30 min for services were 3.88 times more likely to be satisfied (AOR = 3.88; 95% CI: 1.26–11.97). Overall, the ART programs performance evaluation among adult HIV/AIDS patients was evaluated at 74.8%, based on factors such as resource availability, healthcare providers’ adherence to national guidelines, and patient satisfaction (Table 8).

**Table 8:** Factors associated with satisfaction among Adult HIV/AIDS patients at Woldia General Hospital, Ethiopia (*n* = 402).

Variables	Category	Satisfaction		COR; (95%;CI)	AOR; (95%;CI)
		Yes	No		
Age	18–25	22	10	1	1
	26–35	129	39	1.5(0.65–3.44)	1.02 (0.25–4.13)
	36–45	105	29	1.64(0.7–3.86)	1.27 (0.29–5.45)
	≥ 46	56	12	2.12(0.8–5.6)*	1.06 (0.2–5.46)
Sex	Male	135	46	0.72(0.45–1.16)	1.15(0.6–2.18)
	Female	177	44	1	1
Residence	Urban	243	79	0.49 (0.25–0.97)	0.58(0.13–2.49)
	Rural	69	11	1	1
Educational status	No formal education	72	13	1	1
	Primary education	81	19	0.77(0.35–1.66)	0.92(0.34–2.48)
	Secondary education	128	45	0.51(0.26–1.01)*	0.89(0.31–2.52)
	Diploma and above	31	13	0.43(0.17–1.03)*	0.62(0.15–2.51)
Occupational status	Unemployed	37	8	1	1
	Government Employed	34	14	0.52(0.19–1.4)	0.69(0.2–2.35)
	Merchant	34	14	0.52(0.19–1.4)	0.47(0.14–1.59)
	Farmer	38	8	1.03(0.34–3)	0.27(0.04–1.57)
	Daily labor	44	15	0.63(0.24–1.66)	0.55(0.18–1.67)
	Driver	13	7	0.4 (0.12–1.32)*	0.55(0.12–2.38)
	House wife	99	18	1.18(0.47–2.96)	0.88(0.29–2.69)
	Student	13	6	0.46(0.14–1.6)	0.51(0.09–2.79)
Level of income	500–1200	106	17	1	1
	1,201–2400	104	31	0.53(0.28–0.1.03)*	0.58(0.24–1.36)
	2,401–8000	102	42	0.38(0.2–0.73)*	0.52(0.19–1.39)
Waiting time in minutes	10–29	130	16	5(1.79–13.89)*	3.88(1.26–11.97)*
	30–60	169	66	1.57(0.63–3.97)	1.35(0.48–3.76)
	61–120	13	8	1	1
Disclosed HIV status	Yes	295	73	4 (1.96–8.3)	3.25(1.46–7.26)*
	No	17	17	1	1
CD4 done every 6 month	Yes	236	62	1.4(0.84–2.35)	1.08(0.61–1.94)
	No	76	28	1	1
Having drug side effect	Yes	32	15	1	1
	No	280	75	1.75 (0.9–3.39)	2.01(0.96–4.23)

*shows statistical association.

### Observation of patient provider interaction

#### Clinical observation

During data collection, physician-client interactions were observed over four sessions—two in the morning and two in the afternoon across 2 days. In every session, the physician greeted all patients. Each of the 10 patients was asked about their complaints, and for the eight patients eligible for physical examination and laboratory tests, all necessary examinations were conducted, and relevant lab tests were ordered. Throughout all sessions, the physician communicated using simple and clear language. All eight patients eligible for opportunistic infection diagnosis and treatment underwent required laboratory tests, including hematology and chemistry, and received appropriate treatment. The remaining patients were diagnosed and treated clinically. No tests for HCV or HBV were ordered for any patient.

Additionally, two sessions—one in the morning and one in the afternoon—focused on nurse-client interactions. During these sessions, all 10 patients were asked about any health concerns. Among the 10 patients eligible for malnutrition assessment, only eight underwent anthropometric measurements. Nurses consistently used simple and clear language while communicating. Out of the 10 patients eligible for CD4 and viral load testing, only eight had their tests ordered. Furthermore, all patients were screened for tuberculosis and questioned about drug side effects.

### Adherence unit preparation counseling

Nurses and case managers conducted counseling and adherence sessions. A dedicated counseling room was available, ensuring confidentiality during sessions. However, there was no separate counseling space in the pharmacy. A total of six client-healthcare worker interaction sessions were held, with 10 observations conducted overall.

All patients received counseling on their illness and the lifelong necessity of taking medication. They were also encouraged to maintain a positive outlook on life. Counseling covered drug dosage, frequency, adherence, potential mild and severe side effects, and the importance of seeking urgent medical attention if needed. Patients were advised to avoid alcohol, Khat, and drug use, as well as to practice safer sex by using condoms; however, condom use was not demonstrated to any patient.

Additionally, all patients were given guidance on proper nutrition, safe drinking water, and the benefits of disclosing their status. However, none were advised on using bed nets. To minimize the Hawthorne effect, the first and last three observations were excluded from the analysis.

### Over all judgment matrix

The overall judgment matrix had shown the program implementation level was 74.8% which was well implemented. The high score was on availability of resources 88.4% followed by satisfaction of clients 77% and compliance of health care workers 66% (Table 9).

**Table 9:** The overall judgment matrix and analysis to evaluate the process of adult ART program at Woldia General Hospital, Ethiopia, 2020.

Sub dimension	Expected%	Weight%	Observed	Result%
Availability	100	30	25.9	26.52 %
Compliance	100	50	33.6	32.94%
Satisfaction	85	20	14.8	15.4%
Total	295	100	74.3	74.8 (well implemented but needs improvement)

## Discussion

The overall implementation of the ART program for adult HIV/AIDS patients at Woldia General Hospital was assessed at 74.8%. Key components included resource availability (88.4%), healthcare workers’ compliance with national guidelines (66%), and patient satisfaction with services (77%). According to the evaluation criteria, the programs implementation requires improvement. The availability of resources recommended by national guidelines was 88.4%, which was lower than findings from an evaluation at Felege Hiwot Referral Hospital. While most essential resources—such as trained staff, laboratory facilities, and medications—were consistently available, there was a noted shortage of Cotrimoxazole (21). In contrast, an evaluation of the pediatric ART program in Addis Ababa reported 91.6% resource availability (23). Similarly, a study in Brazil found that funding and resources for ART services, including medicines and HIV-specific tests, were consistently high at approximately 90%. Essential medicines for opportunistic infection prophylaxis, biochemical tests for monitoring drug toxicity, and simple imaging tests were available within 2 weeks in 80% of services (24). The differences in resource availability across settings could be attributed to variations in evaluation periods and locations, as well as the withdrawal of many NGOs that previously supported the program. However, the resource availability at Woldia General Hospital was higher than that of an evaluation of the PMTCT program at Agaro Health Centre, where availability was 77.4% (25). This difference may be due to hospitals having better diagnostic facilities and more specialized healthcare personnel.

Regarding compliance with national guidelines, the hospital scored 66%, which was lower than the 81.6% compliance rate observed in an evaluation of the pediatric ART program in Addis Ababa (23). One possible reason for this discrepancy is patient load—currently, a large number of patients are seeking HIV/AIDS care and treatment, and many laboratory tests are no longer provided free of charge. As a result, most tests are not ordered unless the patient is visibly ill.

This evaluation found that 82.4% of eligible patients were receiving Cotrimoxazole Prophylactic Therapy (CPT). This percentage was higher than the 45.9% reported in an evaluation at Felege Hiwot Referral Hospital (21).

A possible explanation for this difference could be the changes in CPT eligibility criteria. The 2008 national guideline, which was in use during the Felege Hiwot evaluation in 2009, recommended CPT for patients with a CD4 count ≤ 200 in stages I and II and for all patients in stages III and IV, regardless of CD4 count. In contrast, the current national guideline recommends CPT for patients with a CD4 count ≤ 350 in stages I and II and for all patients in stages III and IV, regardless of CD4 count, as well as improved CPT availability.

Regarding patient record documentation, 74% of patients had their addresses recorded, which was higher than the 41.9% documented in the Felege Hiwot Referral Hospital evaluation (21). This improvement may be due to increased awareness of data utilization for evidence-based decision-making, although data use remains suboptimal (28).

In terms of laboratory testing, the evaluation found that only 28% of patients were tested for diabetes mellitus (DM), 42% for hypertension (HTN), 96.6% had baseline hematocrit (HCT) tests, and 82% underwent baseline urine analysis. Additionally, 94% had baseline renal function tests (RFT) and liver function tests (LFT). However, screening rates for hepatitis B (HBV) and hepatitis C (HCV) were significantly lower, at 21 and 11%, respectively. These findings fall short of the 2017 national guideline, which recommends screening all patients at baseline for DM, HTN, anemia, urine analysis, RFT, LFT, HBV, and HCV. The low screening rates may be attributed to the fact that these tests are no longer provided for free, leading to their omission unless clinically necessary. Additionally, many HIV/AIDS patients face financial constraints that limit their ability to pay for these tests.

Two key informants mentioned that most laboratory tests were not free, except for CD4 and viral load tests, and many HIV/AIDS patients faced financial difficulties in affording these tests. As a result, tests were only ordered when patients were visibly ill.

In the evaluation of malnourished patients, only 56.6% received treatment for malnutrition, which was below the national guideline that mandates nutritional treatment for all malnourished patients. A possible reason for this low implementation could be the unavailability of Plumpy’Nut, which had expired at the national level.

Regarding cervical cancer screening, only 33.3% of eligible patients were screened, falling short of the national guideline, which recommends screening all female HIV/AIDS patients over 30 years old. The likely cause of this low screening rate was a shortage of Pap smear reagents, as confirmed by two key informants.

Among INH-eligible patients, only 50% were receiving INH treatment, which was lower than the national guideline recommendation that all eligible patients should receive INH.

One key informant noted that some patients refused to take INH, believing that they were not affected by tuberculosis (TB).

In this evaluation, 77% of HIV/AIDS patients reported being satisfied with the services provided. The specific satisfaction rates for various aspects of care were as follows: courtesy of healthcare workers (83%), availability of opportunistic infection (OI) drugs (69%), competency of healthcare workers (88.5%), counseling services (85%), hospital cleanliness (83%), suitability of the waiting area (70%), waiting time (71%), laboratory services (78%), and confidentiality during examinations (85%). Factors associated with patient satisfaction included the ability to disclose their sero status and a waiting time of less than 30 min.

These findings align with studies conducted in different locations, such as Dilchora Hospital in Dire Dawa (77%) (29), Midre Genet Hospital in Tigray (75.2%) (27), and health centers in Gondar town (75.4%) (26).

However, the satisfaction rate in this evaluation was higher compared to studies conducted in Vietnam (42.4%) (30), private hospitals in Nigeria (41.4%) (31), Zewuditu Memorial Hospital in Addis Ababa (38.6%), West Wollega (57.2%) (20), Hossana Town in southern Ethiopia (70.1%) (32) and public hospitals in Nigeria (71.5%) (31). The variation in satisfaction levels may be attributed to differences in healthcare settings and resource availability, as hospitals generally have better diagnostic services, medical personnel, and overall healthcare provision compared to health centers.

Conversely, this study reported a lower satisfaction rate compared to findings from Sokoto Hospital in Nigeria (99.6%) (33), Cameroon (91.2%) (34), Ethiopian public health centers (89%) (35), Addis Ababa public hospitals (85.5% for laboratory services) (36), Zewuditu Memorial Hospital (84.7% for pharmaceutical services) (37), and health centers in Tigray (89.6%) (38). The higher satisfaction rates in these studies could be due to better healthcare infrastructure and greater access to free medications.

Similarly, this evaluation showed that among the indicators used to assess satisfaction, the confidentiality of examinations and competency of health care workers had high satisfaction scores. However, the availability of drugs had a low satisfaction rate. These findings were consistent with studies done in Cameron (34) and Ethiopia public health centres (35).

Additionally, this evaluation revealed that individuals who disclosed their sero status were 3.25 times more likely to be satisfied compared to those who did not. This finding was similar to a study conducted in Hossana Town (32). A possible explanation is that disclosure allows patients to share their concerns, fostering a sense of security and increasing satisfaction (39).

Furthermore, respondents who waited less than 30 min for services were 3.8 times more likely to be satisfied than those who waited over an hour. This outcome was consistent with a study on ART laboratory services in Addis Ababa (36). The likely reason for this is that prolonged waiting times can cause psychological distress, leading to increased stress and overall dissatisfaction (40).

### Barriers to ART services implementation at Woldia General Hospital

One of the key informants mentioned that laboratory tests such as CBC, OFT, HBV, and HCV were not conducted in accordance with ART guidelines before initiating treatment unless there was a specific indication. This was because these tests were not free, and many patients could not afford them.

Three key informants highlighted that one of the major barriers to ART services was the availability of opportunistic infection (OI) drugs, which were not provided free of charge. Since most patients could not afford these medications, this resulted in poor treatment outcomes and was considered a failure of the ART program.

Another three key informants pointed out that budget constraints made it difficult to track patients who had been lost to follow-up. Case managers often had to use their own money to locate these individuals.

Additionally, three key informants stated that viral load testing was not available at their facility. Instead, samples had to be sent to Dessie Referral Hospital, leading to significant delays in receiving test results. Many patients expressed dissatisfaction with these delays.

Two key informants noted that limited space in examination rooms was another challenge in providing ART care services. In some cases, two healthcare professionals had to examine two patients in the same room, compromising privacy.

Lastly, three key informants reported that the unavailability of Plumpy’Nut (a therapeutic food) and national shortages of HIV test kits made it difficult to manage malnourished patients and properly screen vulnerable individuals.

### Strengths and Limitations of the evaluation

A case study design with a mixed-methods approach was applied in this evaluation to maximize the strength of the evaluation. This evaluation focused on the ART program. Which is the program of global concern. The ART patients deserves priority for any program evaluation, research and projects which can improve their quality of life.A potential limitation of this evaluation may be the presence of social desirability bias, where patients may have given overly positive responses about the hospital services. Additionally, observation bias could have influenced the results.

## Conclusion

The overall implementation of the program was very good.However, healthcare workers’ adherence to national guidelines and HIV/AIDS patients’ satisfaction with services fell short of national targets. While there was no shortage of trained healthcare workers, ART drugs, anti-TB drugs, cotrimoxazole, or INH. There was a scarcity of opportunistic infection (OI) drugs, Plumpy’Nut and additional medications like ciprofloxacin and cloxacillin. Furthermore, viral load tests and HIV test kits were unavailable, posing significant barriers to the successful execution of ART services.

Several essential laboratory tests, including baseline OFT, CBC, cervical cancer screening, and HBV and HCV tests, were not conducted in accordance with national guidelines. Additionally, issues with chart management and incomplete patient records were identified. Factors associated with patient satisfaction were disclosing HIV status and experiencing a waiting time of less than 30 minutes.

To enhance program implementation, the hospital is expected ensure the availability of necessary resources, and healthcare providers are also expected to adhere to national guidelines while properly managing patient records. Additionally, patients need to be encouraged to disclose their HIV status, and the hospital need to develop strategies to reduce waiting times, ultimately improving patient satisfaction.

## Data Availability

The original contributions presented in the study are included in the article/supplementary material, further inquiries can be directed to the corresponding author.

## Glossary

AFB: Acid Fast Bacilli
AIDS: Acquired Immuno Deficiency Syndrome
ART: Anti Retro Viral Therapy
ARV: Anti Retro Viral CBC Complete Blood Count
CD4: Cluster of Differentiation
CDC: Center for Disease Control
CPT: Cotrimoxazole Preventive Therapy
DM: Diabetes Melites
ETB: Ethiopian Birr
FMOH: Federal Ministry of Health
HAART: Highly Active Anti Retro Viral Therapy
HBV: Hepatitis B Virus
HCT: Haematocrit
HCV: Hepatitis C Virus
HIV: Human Immunodeficiency Virus
HTN: Hypertension
INH: Isoniazid
LFT: Liver Function Test
NGOs: Non-Governmental Organizations
NVP: Nevirapine
OIs: Opportunistic Infections
RFT: Renal Function Test
STI: Sexually Transmitted Infections
TB: Tuberculosis
TDF: Tenefovir Disoproxil Fumarate
UA: Urine Analysis
WHO: World Health Organization
